# The HIV care continuum and determinants of antiretroviral therapy initiation among transgender women in France (1997–2023): a longitudinal cohort study

**DOI:** 10.1016/j.lanepe.2026.101820

**Published:** 2026-08-20

**Authors:** Juliette Hemery, Pierre Tattevin, Esaïe Marshall, Margot Annequin, Thibault Chiarabini, Nathalie Dournon, Anaenza Freire Maresca, Jade Ghosn, Valentina Isernia, Florence Michard, Segolene Perrineau, Giovanna Rincon, Elisabeth Rouveix, Bruno Spire, Pierre de Truchis, Sophie Grabar

**Affiliations:** a Institut Pierre Louis d’Epidémiologie et de Santé Publique, Inserm, Sorbonne Université, Paris, France; b Service des Maladies Infectieuses et Emergentes, Hôpital Pontchaillou, Rennes, France; c Inserm, IRD, SESSTIM, Sciences Economiques & Sociales de la Santé & Traitement de l’Information Médicale, ISSPAM, Aix Marseille Univ, Marseille, France; d AP-HP, Hôpital Saint-Antoine, Paris, France; e AP-HP, Hôpital Raymond Poincaré, Paris, France; f AP-HP, Hôpital Ambroise Paré, Paris, France; g UFR Santé Simone Veil, Université Paris Saclay, Garches, France; h AP-HP, SMIT, Hôpital Avicenne, Bobigny, France; i AP-HP, CSAPA, Hôpital Fernand Widal, Paris, France; j AP-HP, SMIT, Hôpital Bichat, Paris, France; k Université Paris Cité, Inserm, IAME, Paris, France; l Association Acceptess-T, Paris, France; m COREVIH Ile de France Ouest, France

**Keywords:** France, HIV, HIV care continuum, ART initiation, Transgender women, Viral suppression

## Abstract

**Background:**

Studies on transgender women living with HIV (TWH) are scarce in Europe. We aimed to describe TWH and their care continuum during years 1997–2023 in France, focusing on determinants of time to antiretroviral therapy (ART) initiation.

**Methods:**

In this longitudinal cohort study, we included treatment-naïve TWH with immuno-virological data from the ANRS CO4 French Hospital Database on HIV (FHDH). HIV care continuum outcomes, ART and viral suppression rates, and time intervals between HIV diagnosis, ART initiation, and viral suppression, were analyzed across three calendar periods (1997–2004, 2005–2012, and 2013–2023). Factors associated with time to ART initiation were assessed for each period using Cox models adjusted for age, geographic origin, year and region of enrollment, time between diagnosis and enrollment, and baseline immuno-virological status.

**Findings:**

801 TWH entered care between January 1, 1997 and December 31, 2023, mostly originating from Latin America (65%, 522/801), with a median age of 31 years [IQR, 27–36]. Between 1997–2004 and 2013–2023, time to ART initiation after entering care decreased from 3.9 [0–31] to 0.5 [0.2–1.2] months. More advanced HIV disease was associated with faster ART initiation until 2012 (hazard ratio [HR]: 3.17, 95% CI 2.14–4.68 for CD4 <200/mm^3^ or AIDS in 2005–2012). In 2013–2023, baseline viral load ≥100,000 was associated with faster ART initiation (HR: 1.45, 95% CI 1.14–1.86), whereas originating from Latin America (HR: 0.68, 95% CI 0.50–0.91) or other countries outside France (HR: 0.65, 95% CI 0.45–0.95) was associated with delayed ART initiation. Time to viral suppression after ART initiation decreased from 6.2 [3.0–27.9] in 1997–2004 to 2.3 [1.1–5.7] months in 2013–2023. Over the same periods, TWH on ART with viral suppression increased from 74.7% (109/146) to 95.5% (315/330).

**Interpretation:**

Despite major improvements in the HIV care continuum for TWH in France, including faster ART initiation and viral suppression reaching UNAIDS 95% targets in 2013–2023, disparities by geographic origin have emerged, showing a delay in ART initiation among foreign-born TWH. Given that most TWH are foreign-born, targeted strategies and policies are needed to ensure equitable and timely access to HIV care.

**Funding:**

ANRS-MIE and INSERM.

**Research in context**

**Evidence before this study**

Transgender women are recognized as a key population in the global HIV epidemic, as they are disproportionately affected by HIV infection worldwide. It is important to move beyond cross-sectional descriptions of the care continuum and adopt a longitudinal approach to provide a better understanding of the epidemic in this population, especially in high-income countries with broad access to HIV testing and treatment. We searched PubMed from database inception to May 10, 2026, for studies published on transgender women living with HIV (TWH) and the HIV care continuum, using the following terms: “HIV” AND (“transgender women” OR “transfeminine” OR “male to female”) AND (“HIV care continuum” OR “retention in care” OR “antiretroviral therapy” OR “viral suppression”). Existing evidence, mostly cross-sectional studies conducted in North America, has shown poorer HIV care outcomes in TWH. Only one study has described the HIV care continuum in TWH in Europe, without analyzing delays between key stages or their determinants.

**Added value of this study**

Using nearly three decades of data from a large, nationally representative cohort, this study provides the first comprehensive longitudinal assessment of the HIV care continuum in TWH, examining rates and delays between key stages, along with determinants of time to ART initiation. Despite overall improvements, with shortened delays and ART coverage and viral suppression reaching the 95% UNAIDS targets, longer time to treatment initiation emerged among foreign-born TWH compared to those born in France, highlighting structural inequities in timely HIV care even within a universal health system.

**Implications of all the available evidence**

Persistent disparities affecting foreign-born TWH suggest that universal health coverage alone does not guarantee timely ART initiation. Tailored strategies addressing structural and migration-related barriers are essential to ensure equitable HIV care for all TWH, alongside maintaining and strengthening inclusive policies that support access to care for migrants.

## Introduction

Transgender women (TW) are disproportionately affected by HIV, with an estimated prevalence of approximately 20% worldwide and 7% in Europe.^1^^,^^2^ Transgender women living with HIV (TWH) face multilevel barriers to access and engagement in HIV care, such as stigma, discrimination, socioeconomic marginalization, mental health challenges and limited access to healthcare professionals trained in gender identity issues.^3^, ^4^, ^5^, ^6^, ^7^ These obstacles not only delay entry into care, but also hinder their ability to start and remain on antiretroviral therapy (ART). Some cross-sectional studies have reported lower retention in care and ART uptake in TWH compared to other people living with HIV (PWH),^8^, ^9^, ^10^, ^11^ which sometimes results in a lower chance of achieving viral suppression.^9^^,^^12^^,^^13^

Understanding the HIV care continuum among TWH is essential to identify gaps and guide interventions. However, most existing studies on this topic are cross-sectional, unable to provide any information on the time between stages of the HIV care continuum. Additionally, most of the research has been conducted in North America, where health coverage and population contexts differ from Europe. Only one study from Europe, in the Netherlands,^14^ has been conducted on the HIV care continuum in TWH, and none in France, where there is universal health coverage, including access to HIV care for undocumented migrants via the state medical aid (AME). Moreover, to our knowledge, no longitudinal study has ever focused on determinants of time to ART initiation after care entry in TWH.

This study aimed to describe treatment-naïve TWH in France from 1997 to 2023, using data from a large nationwide cohort, the ANRS CO4 French Hospital Database on HIV (FHDH), and to analyze trends in HIV care continuum outcomes and time intervals (diagnosis, linkage to care, initiation of ART, and viral suppression) across three calendar periods: 1997–2004, 2005–2012, and 2013–2023, focusing on determinants of time to ART initiation.

## Methods

### Data source

The ANRS CO4 FHDH is a national open hospital cohort that follows people living with HIV (PWH) receiving care in over 180 public hospitals across France since 1989. Participants with documented HIV-1 or HIV-2 infection receiving care at a participating FHDH center were eligible after providing written consent. This cohort provides a representative sample of PWH in care in France, covering about 65% of those engaged in care nationwide in 2023 (more details at: https://anrs-co4.fhdh.fr).^15^ Over 107,000 PWH were followed across 166 different hospitals in 2023. Sociodemographic, clinical, biological and therapeutic data were collected from medical records. Over time, data collection systems evolved with routine validation procedures ensuring quality and continuity of data capture, while data completeness remained high for HIV-related variables.

### Classification of sex and gender identity

Prior to 2013, sex was collected as a binary variable (“male” or “female”) in medical records and in the ANRS CO4 FHDH cohort. A “transgender” category was introduced in 2013, but did not specify the sex assigned at birth nor the current gender identity (i.e., male-to-female or female-to-male). Transgender participants enrolled before 2013 could be reclassified as “transgender” during follow-up visits after the category became available in 2013.

Using this variable, a total of 1686 transgender participants were identified in the FHDH among PWH enrolled between 1997 and 2023 (Supplementary Figure S1). To further identify transgender women (TW; male-to-female), we applied a predefined algorithm combining diagnostic and therapeutic information available in the cohort. Male participants with a diagnosis of “*gender identity disorder*” (ICD-10 code ‘F64’) were classified as TW. Among transgender participants, those whose mode of HIV acquisition was “Men who have sex with men” (MSM), who received feminizing hormone therapy (ATC codes ‘G03CA03’ for estradiol, and ‘G03HB01'/'G03HA01’ for cyproterone), or who were diagnosed with sex-specific malignant neoplasms of the male genital organs (ICD-10 code ‘C60–C63’ including penis, prostate and testis) were also classified as TW.

A similar procedure was used to identify transgender men. Other transgender participants were unclassified (Supplementary Figure S2).

### Study population

We selected treatment-naïve transgender women with HIV (TWH) who were enrolled and then followed between January 1, 1997, and December 31, 2023, in the ANRS CO4 FHDH cohort. Inclusion criteria were: (i) being ART-naïve (never received ART and with a viral load (VL) > 200 copies/mL) at enrollment; (ii) having available baseline VL and CD4 cell counts at enrollment; (iii) having at least two subsequent VL measurements after initiating ART, ensuring a minimum of three documented VL measurements overall except in cases of death (n = 2). TWH receiving treatments other than dual or triple ART regimens (n = 2), or who acquired HIV through injecting drug use (IDU, n = 10) were excluded due to small sample size limiting reliable statistical adjustment or subgroup analyses.

### Statistical analysis

The characteristics of TWH were described across three calendar periods defined by different treatment recommendations and availability in France: 1997–2004, 2005–2012 and 2013–2023. The first period (1997–2004) was characterized by early combination ART (cART), with mainly triple-drug regimens associated with poor tolerability, and treatment initiation driven by CD4 cell count thresholds, initially <200 and later <350 cells/mm^3^. The second period (2005–2012) corresponded to an era of better tolerability and more effective cART, with French recommendations progressively expanding treatment initiation to CD4 counts up to <500 cells/mm^3^. The third period, 2013–2023, was characterized by the implementation of the “Treat All” policy, recommending immediate ART initiation regardless of CD4 count. These guidelines were implemented in France earlier than the 2015 WHO recommendation.^16^ Demographic, immunovirological, and clinical characteristics at enrollment were compared across calendar periods using chi-square and Kruskal–Wallis tests.

The date of enrollment in FHDH was considered the baseline. For both VL and CD4 count, the baseline value was the measurement closest to the enrollment date, within ±3 months for VL and from 6 months before to 3 months after for CD4 count. If treatment was initiated within this period, only measurements documented up to 15 days after treatment initiation were considered for both VL and CD4 count.

Time intervals along the HIV care continuum were defined as follows: (1) the time from the first positive HIV serology to care entry (i.e., date of enrollment in FHDH); (2) the time from care entry to ART initiation; and (3) the time from ART initiation to virologic suppression, defined as two consecutive VL measurements below 200 copies/mL, a threshold chosen to account for changes in assay detection limits over time. Median time intervals and cumulative incidence curves were estimated using the Kaplan–Meier method and compared across calendar periods, using the log-rank test. Follow-up was censored at the date of last follow-up (defined as the last recorded medical visit), death, or loss to follow-up, whichever occurred first. Lost to follow-up (LTFU) was defined as an interval of ≥18 months between the last recorded medical visit and the most recent database update (December 2023).

**Fig. 1:**
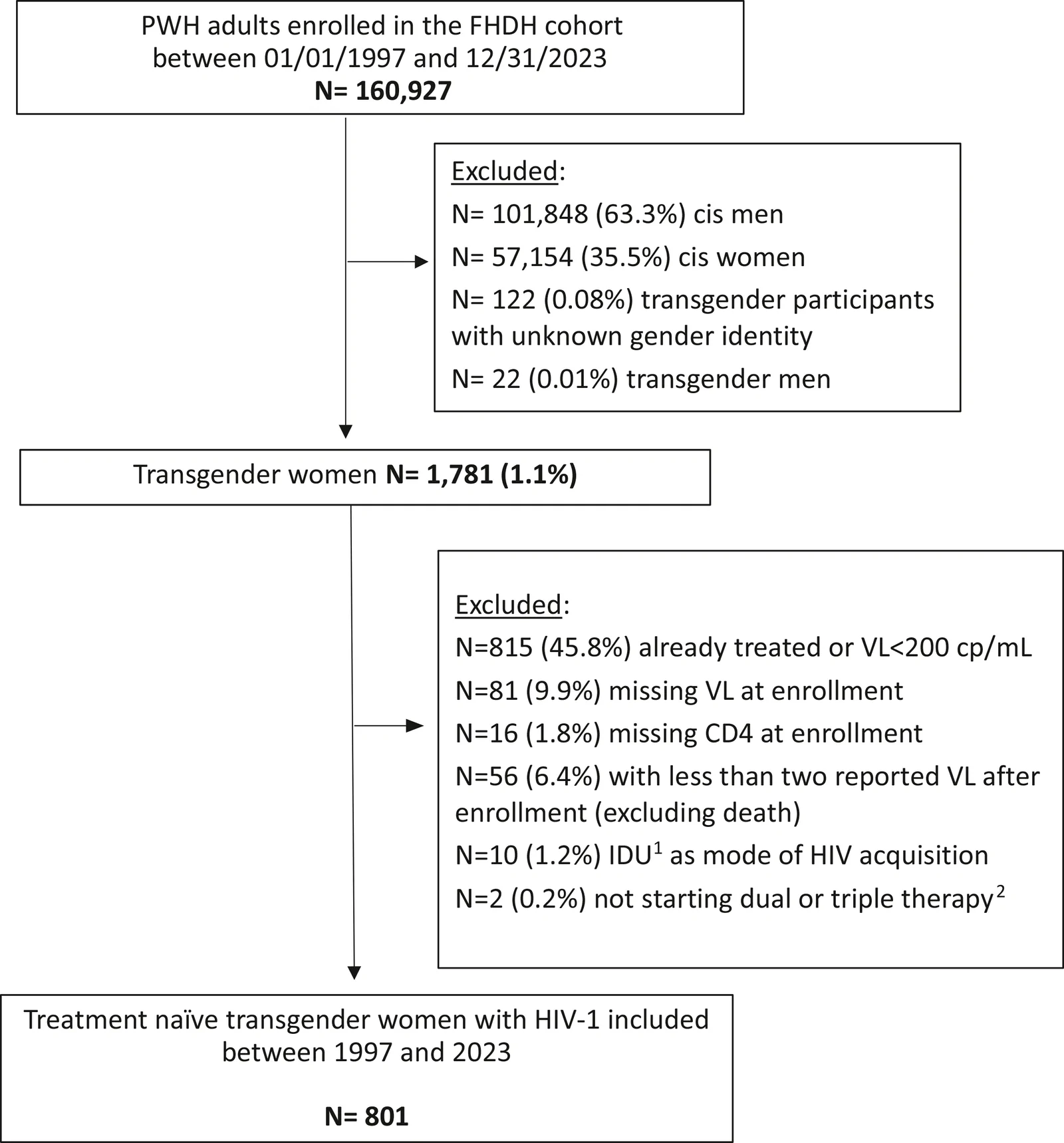
**Flow chart of the population selection.**^1^IDU: Injecting drug user. ^2^Among those who started antiretroviral therapy (ART). Abbreviations: PWH, people living with HIV; FHDH, French hospital database on HIV; VL, viral load.

For each calendar period, we estimated the proportion of transgender women receiving ART and achieving virologic suppression. To ensure sufficient follow-up for these outcomes to occur, analyses were restricted to participants enrolled up to one year before the end of each calendar period. Sensitivity analyses were performed including the excluded TWH (Fig. 1).

Factors associated with time to ART initiation after care entry were assessed using Cox proportional hazards models for each calendar period. Continuous variables were categorized for clinical interpretability. There were no missing data for variables included in the models. Models were adjusted for age (18–29, 30–39, ≥40), geographic origin (France, Latin America, other countries), region of inclusion (Paris region, other regions), baseline immunological status at care entry (<200 CD4 cells/mm^3^ or AIDS, 200–350 CD4 cells/mm^3^, ≥350 CD4 cells/mm^3^ or primary HIV infection) and VL at care entry (<100,000 copies/mL, ≥100,000 copies/mL), time between diagnosis and care entry (<1 month, 1–3 months, >3 months), and year of enrollment. The proportional hazards assumption was verified using Schoenfeld residuals.

Analyses were performed using SAS 9.4 and R 4.2.2. Statistical significance was defined as a p-value <0.05.

### Ethics approval

The ANRS CO4 FHDH was first authorized by the French Data Protection Authority (Commission Nationale de l’Informatique et des Libertés – CNIL) in 1991. The research authorization was updated to comply with new regulations, including the General Data Protection Regulation. The ANRS CO4 FHDH cohort was approved by the CEREES (Comité d’Expertise pour les Recherches, les Études et les Évaluations dans le domaine de la Santé) in 2018, and as a hospital data warehouse authorized to conduct research by the CNIL in 2021. All participants included provided written informed consent for the use of their data.

### Role of the funder

The funder of the study had no role in study design, data collection, data analysis, data interpretation, or writing of the report.

## Results

Among the 160,927 PWH included between January 1, 1997, and December 31, 2023, in the FHDH cohort, 1781 were identified as transgender women (TW), 22 transgender men and 122 unclassified (Fig. 1). Of these, 801 TW fulfilled the study inclusion criteria and were followed for a median of 10 years (IQR, 5–16, Table 1).

**Table 1:** Characteristics of the 801 transgender women with HIV in the FHDH cohort by calendar period.

	Overall period (1997–2023) N = 801	1997–2004 N = 204	2005–2012 N = 245	2013–2023 N = 352	P-value
At enrollment in FHDH (care entry)					
Age (years)^a^	31 (27–36)	32 (27–36)	31 (26–36)	31 (27–36)	0.7799
Geographic origin					0.1927
Latin America	522 (65.2)	138 (67.6)	164 (66.9)	220 (62.5)	
France	182 (22.7)	40 (19.6)	61 (24.9)	81 (23.0)	
Africa	44 (5.5)	9 (4.4)	10 (4.1)	25 (7.1)	
Other countries/region of the world	53 (6.6)	17 (8.3)	10 (4.1)	26 (7.4)	
Included in Paris region	559 (69.8)	160 (78.4)	170 (69.4)	229 (65.1)	**0.0041**
Year of enrollment	2011 (2004–2017)	2001 (1999–2003)	2009 (2007–2011)	2018 (2016–2020)	**<0.0001**
HIV acquisition group					0.8700
Sexual transmission from a cis-man	788 (98.4)	200 (98.0)	241 (98.4)	347 (98.6)	
Other or unknown	13 (1.6)	4 (2.0)	4 (1.6)	5 (1.4)	
Immuno clinical status					**0.0002**
<200/mm^3^ CD4 or AIDS	183 (22.8)	61 (29.9)	46 (18.8)	76 (21.6)	
200–350/mm^3^ CD4	159 (19.9)	51 (25.0)	55 (22.4)	53 (15.1)	
≥350/mm^3^ CD4 or primary infection	459 (57.3)	92 (45.1)	144 (58.8)	223 (63.4)	
AIDS (CDC C clinical stage)	74 (9.2)	28 (13.7)	18 (7.3)	28 (8.0)	**0.0362**
CD4 (cells/mm^3^)	381 (232–540)	311 (182–512)	394 (266–548)	410 (250–547)	**0.0102**
CD4/CD8 ratio	2.3 (1.5–3.8)	2.9 (1.8–4.6)	2.1 (1.5–3.0)	2.2 (1.5–4.0)	**0.0061**
HIV RNA copies/mL					**0.0093**
200–5000	106 (13.2)	26 (12.7)	31 (12.7)	49 (13.9)	
5000–100,000	391 (48.8)	99 (48.5)	141 (57.6)	151 (42.9)	
≥100,000	304 (38.0)	79 (38.7)	73 (29.8)	152 (43.2)	
HBV-positive	30 (3.7)	12 (5.9)	7 (2.9)	11 (3.1)	0.1741
HCV-positive	20 (2.5)	9 (4.4)	3 (1.2)	8 (2.3)	0.0919
Follow-up					
Type of first ART regimens					**0.0021**
Not receiving ART	11 (1.4)	4 (2.0)	5 (2.0)	2 (0.6)	
Dual therapy	26 (3.2)	14 (6.9)	3 (1.2)	9 (2.6)	
Triple therapy	764 (95.4)	186 (91.2)	237 (96.7)	341 (96.9)	
Duration of follow-up (years)	10 (5–16)	21 (17–23)	13 (11–15)	5 (2–7)	**<0.0001**
Lost to follow-up	197 (24.6)	68 (33.3)	73 (29.8)	56 (15.9)	**<0.0001**
Death during follow-up	17 (2.1)	9 (4.4)	6 (2.5)	2 (0.6)	**0.0093**
Under ART at last follow-up^b^	774 (98.0)	194 (97.0)	235 (98.0)	345 (98.6)	0.4655
HIV RNA <200 cp/mL at last follow-up^b^	726 (91.9)	187 (93.5)	219 (91.3)	320 (91.4)	0.9322

p-value from chi-square or Kruskal–Wallis tests comparing the 3 calendar periods. P-values <0.05 are shown in bold.

ART, antiretroviral therapy; FHDH, French hospital database on HIV.

a Median (IQR) or N (%).

b Among TWH who initiated ART, N = 790.

At enrollment, median age was 31 years (IQR, 27–36). Over 65% (522/801) of TWH originated from Latin America, mostly from Peru (43%, 223/522), Brazil (21%, 109/522) and Ecuador (15%, 80/522, Supplementary Table S1). Most TWH (70%, 559/801) were followed in the Paris region, and 98% (788/801) acquired HIV through sexual intercourse with a cisgender man. About 25% (197/801) of TWH were lost to follow-up (LTFU) during the study period. Participants LTFU were more frequently enrolled in earlier calendar periods and were younger at enrollment compared with those retained in care; other baseline characteristics were similar (Supplementary Table S2).

The age distribution and geographic origin of TWH remained stable across calendar periods. Between 1997–2004 and 2013–2023, the proportion of TWH presenting at an advanced stage of the disease (<200 CD4 cells/mm^3^ or AIDS) decreased from 30% (61/204) to 22% (76/352), while those with baseline VL ≥ 100,000 copies/mL increased from 39% (79/204) to 43% (152/352).

**Fig. 2:**
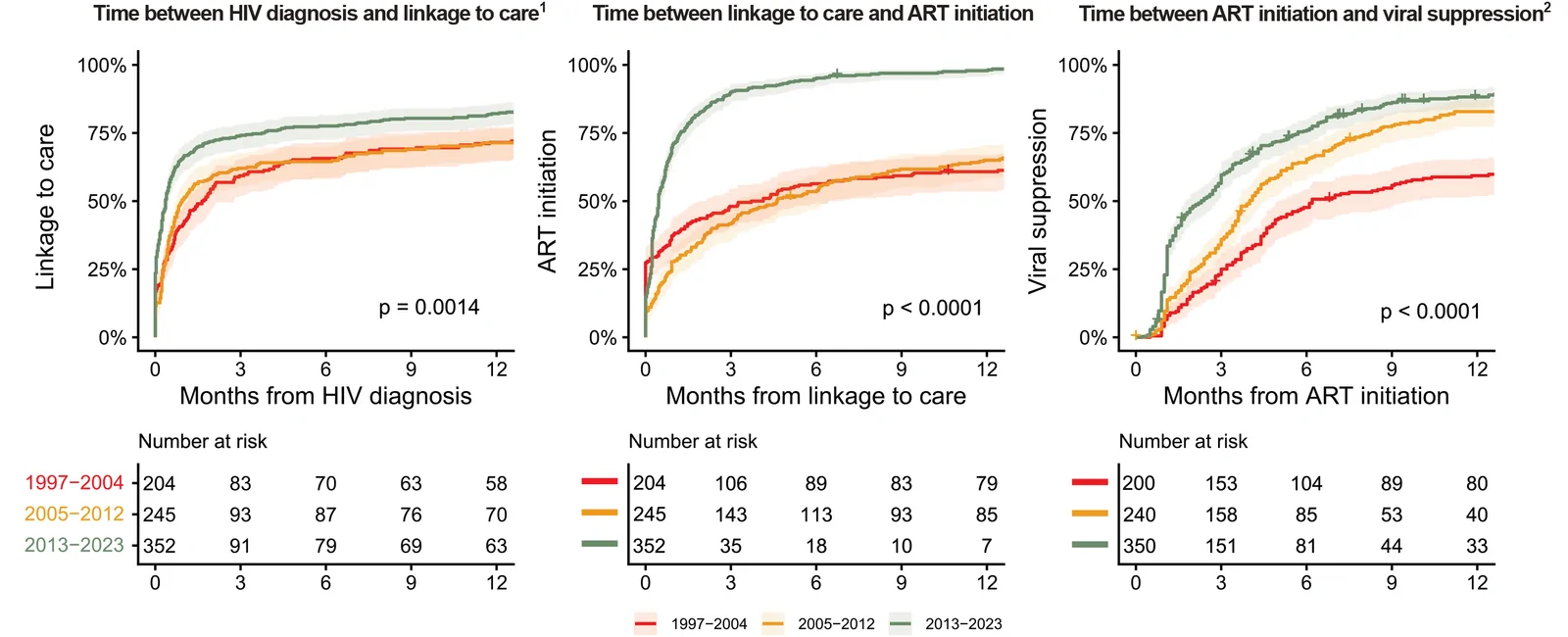
**Evolution of time intervals along the HIV care continuum in transgender women with HIV in France by calendar period.** Results are from Kaplan Meier analyses with 95% CI and p-values from Log-rank tests. ^1^Linkage to care was defined as enrollment in the FHDH cohort. ^2^Viral suppression was defined as two consecutive viral load measurements <200 copies/mL. Only participants who initiated ART were analyzed (N = 790). Abbreviations: ART, antiretroviral therapy; FHDH, French hospital database on HIV.

**Fig. 3:**
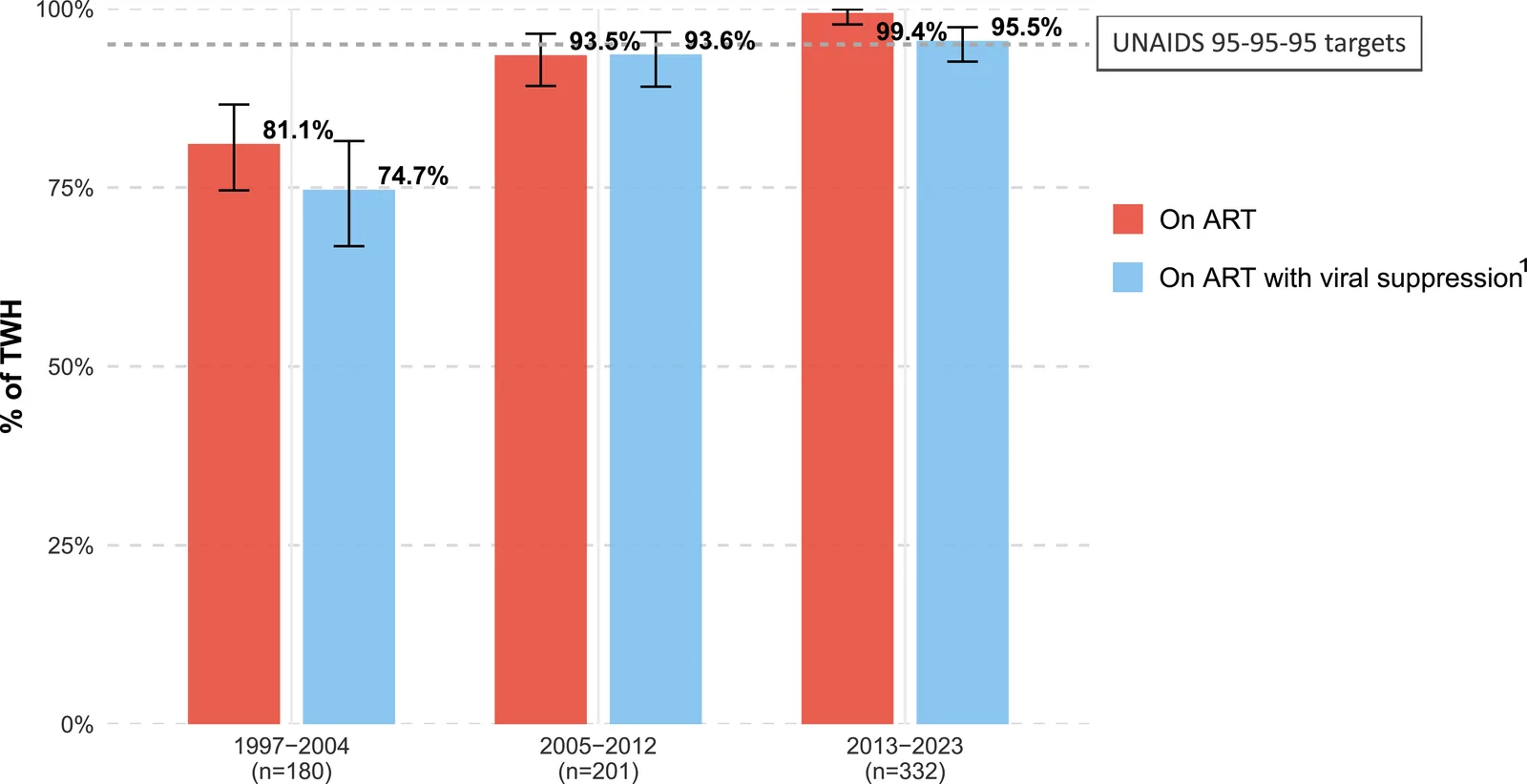
**Proportion of TWH on ART and with viral suppression by calendar period.** Only participants enrolled up to one year before the end of each calendar period were included to allow both events to occur (ART initiation and viral suppression), therefore the number or participants are slightly lower than in Table 1. ^1^Viral suppression was defined as two consecutive viral load measurements <200 copies/mL. Abbreviations: TWH, transgender women living with HIV; ART, antiretroviral therapy.

Changes in time intervals along the HIV care continuum are shown in Fig. 2 and Table 2. Between 1997–2004 and 2013–2023, the median time from HIV diagnosis to care entry decreased from 1.7 (IQR, 0.3–18.4) to 0.4 months (IQR, 0.0–3.8). Over the same periods, the median time from care entry to ART initiation decreased from 3.9 (IQR, 0.0–31.0) to 0.5 months (IQR, 0.2–1.2), and time from ART initiation to viral suppression from 6.2 (IQR, 3.0–27.9) to 2.3 months (IQR, 1.1–5.7). Concurrently, the proportion of TWH on ART with viral suppression increased from 74.7% (109/146) to 95.5% (315/330) (Fig. 3). Sensitivity analyses including the excluded TWH showed similar proportions (Supplementary Table S3). At last follow-up, 98% of TWH (774/790) were still on ART, and 92% (726/790) had a viral load <200 copies/mL (Table 1).

**Table 2:** Evolution of time intervals between stages of the HIV care continuum of TWH by calendar period.

Median (IQR) time intervals in months between:	1997–2004 N = 204	2005–2012 N = 245	2013–2023 N = 352	P-value
HIV diagnosis—Linkage to care^a^	1.7 (0.3–18.4)	1.0 (0.3–17.7)	0.4 (0.0–3.8)	**0.0014**
Linkage to care—ART initiation	3.9 (0.0–31.0)	4.6 (0.9–20.6)	0.5 (0.2–1.2)	**<0.0001**
ART initiation—Viral suppression^b^	6.2 (3.0–27.9)	4.0 (2.1–8.3)	2.3 (1.1–5.7)	**<0.0001**

Estimates from Kaplan Meier and p-values from Log-rank tests comparing the three calendar periods.

Only participants who initiated ART were analyzed for the time interval between ART initiation and viral suppression (N = 790).

TWH, transgender women living with HIV; ART, antiretroviral therapy; FHDH, French hospital database on HIV.

a Linkage to care was defined as enrollment in the FHDH cohort.

b Viral suppression was defined as two consecutive viral load measurements <200 copies/mL.

Factors associated with time to ART initiation changed over time (Table 3). In the first two calendar periods 1997–2004 and 2005–2012, immunological status at care entry was strongly associated with ART initiation, whereas this association was no longer observed in 2013–2023. In 1997–2004, TWH with advanced HIV disease were more likely (HR 4.28, 95% CI 2.80–6.54) to initiate ART earlier compared to those with early HIV disease (≥350 CD4 cells/mm^3^ or HIV primary infection). This association remained substantial in 2005–2012 (HR 3.17, 95% CI 2.14–4.68). A graded association between HIV stage and ART initiation was observed in the first two periods.

**Table 3:** Factors associated with time to ART initiation after care entry in TWH, by calendar periods. Results from multivariable Cox models.

Characteristics at enrollment	1997–2004 N = 204 (200/4)^a^		2005–2012 N = 245 (240/5)^a^		2013–2023 N = 352 (350/2)^a^	
	HR (95% CI)	P-value	HR (95% CI)	P-value	HR (95% CI)	P-value
Year	0.93 (0.86–1.00)	**0.0454**	1.08 (1.02–1.15)	**0.0087**	1.18 (1.14–1.23)	**<0.0001**
Age		**0.0130**		0.2602		0.7079
18–29	0.65 (0.47–0.90)		0.79 (0.59–1.05)		0.90 (0.71–1.16)	
30–39	ref		ref		ref	
≥40	1.11 (0.72–1.73)		0.93 (0.61–1.42)		0.98 (0.71–1.35)	
Origin		0.1454		0.6621		**0.0218**
France	ref		ref		ref	
Latin America	0.75 (0.49–1.15)		0.85 (0.59–1.23)		0.68 (0.50–0.91)	
Other countries	1.14 (0.67–1.94)		0.96 (0.56–1.64)		0.65 (0.45–0.95)	
Region of care		0.9606		0.0554		0.7425
Paris region	ref		ref		ref	
Other regions	1.01 (0.67–1.51)		1.41 (0.99–1.99)		1.04 (0.82–1.32)	
Immuno clinical status		**<0.0001**		**<0.0001**		0.2444
<200/mm^3^ CD4 or AIDS	4.28 (2.80–6.54)		3.17 (2.14–4.68)		0.80 (0.60–1.08)	
200–350/mm^3^ CD4	2.49 (1.69–3.66)		2.38 (1.68–3.37)		1.06 (0.77–1.46)	
≥350 CD4/mm^3^ or primary infection	ref		ref		ref	
Time diagnosis—enrollment		0.5535		0.0589		0.5708
<1 month	ref		ref		ref	
1–3 months	1.09 (0.72–1.67)		1.65 (1.08–2.53)		0.84 (0.56–1.26)	
>3 months	0.88 (0.64–1.21)		1.21 (0.90–1.63)		0.90 (0.69–1.18)	
Viral load		**0.0102**		**<0.0001**		**0.0029**
<100,000 copies/mL	ref		ref		ref	
≥100,000 copies/mL	1.58 (1.11–2.23)		2.55 (1.84–3.54)		1.45 (1.14–1.86)	

Adjusted for all covariates above, for each period. All covariates were complete. HR = Hazard Ratio. P-values <0.05 are shown in bold.

ART, antiretroviral therapy; TWH, transgender women living with HIV.

a Number of events (participants that started ART)/numbers of censored (participants not starting ART).

Viral load (VL) at care entry was associated with time to ART initiation in all calendar periods. TWH with VL ≥ 100,000 copies/mL were significantly more likely to initiate ART earlier compared to those with a VL < 100,000 copies/mL (HRs ranging from 1.45 to 2.55 depending on the period).

Geographic origin was not associated with time to ART initiation in the first two periods. However, in 2013–2023, TWH born in Latin America (HR 0.68, 95% CI 0.50–0.91) or other countries (0.65, 95% CI 0.45–0.95) were less likely to initiate ART early compared to those born in France. Age was only associated in the first period, with younger TWH aged between 18 and 29 years being less likely to start ART earlier compared to those aged 30–39 years (HR 0.65, 95% CI 0.47–0.90).

## Discussion

Using data from a nationwide hospital cohort in France, the ANRS CO4 FHDH, this study provides a longitudinal description of 801 transgender women living with HIV (TWH) in care in France for a 27-year period. Between 1997–2004 and 2013–2023, while TWH sociodemographic characteristics remained stable, their immunological profile improved considerably, with the proportion presenting with advanced HIV disease decreasing by one third. Over the same period, time intervals along the HIV care continuum shortened notably, with median time from care entry to ART initiation decreasing from 3.9 months to 15 days, and time from ART initiation to viral suppression from 6.2 to 2.3 months. Additionally, the proportion of TWH on ART with viral suppression increased from 75% to 96%. Before 2013, time to ART initiation was mainly driven by immunological status, whereas in 2013–2023, disparities based on geographic origin emerged, with TWH born abroad being less likely to initiate ART early than those born in France despite universal access to HIV care.

The sociodemographic profile of TWH in care in France did not change over nearly 3 decades. TWH in France entered care at a relatively young age (median of 31 years), as observed in other studies worldwide.^14^^,^^17^, ^18^, ^19^ A large proportion of TWH were born in Latin America (65%), a pattern consistent with the other few studies on TWH in Europe,^14^^,^^20^ but distinct from North America, where Black TW are more prevalent than Hispanic TW among TWH.^13^^,^^21^, ^22^, ^23^, ^24^ This reflects different structural contexts where migrant status in Europe and race in the US intersect with transgender identity to shape HIV vulnerability. Intersectional frameworks suggest that HIV vulnerability in transgender populations is shaped by interacting structural factors, including cissexism, racism, and xenophobia, which together increase social and health inequities.^4^^,^^23^^,^^25^ In the French context, transgender migrants face barriers to legal gender recognition,^20^ and may experience discrimination related to migration status, contributing to anticipated stigma, mistrust in healthcare, and reduced access to HIV prevention, testing, and care.

Clinical presentation at care entry improved substantially over time, with 63% of TWH presenting with early HIV disease (>350 CD4 cells/mm^3^ or primary infection) in 2013–2023. Similar improvements in CD4 count over time were documented among TWH in New York City between 2007 and 2016.^26^ Nonetheless, a sizeable proportion (22%) of TWH in our cohort entered care with advanced HIV disease (<200 CD4 cells/mm^3^ or AIDS) in 2013–2023, underscoring the need for further research to identify factors associated with late presentation in this key population.

A major contribution of this study is the detailed examination of time intervals along the HIV care continuum among TWH. In the most recent period, time from diagnosis to care entry and from care entry to ART initiation were markedly reduced (to around 15 days), reflecting improved responsiveness of the healthcare system and the implementation of immediate treatment strategies. These findings align with observations from the Athena cohort in TWH in the Netherlands,^14^ and from the general population of PWH in France^27^ and elsewhere, but have rarely been documented specifically in TWH over such a long time span. Our study reported a median time to viral suppression of 2.3 months, slightly longer than the 38 days reported in the Athena cohort,^14^ likely due to our more conservative definition of virologic success. This does not detract from the overall improvements observed.

The determinants of time to ART initiation evolved over time in ways that mirror major shifts in HIV treatment policy. After 2012, immunological status was no longer associated with time to ART initiation, a change mainly driven by the 2013 “treat all” guidelines in France,^28^ recommending ART initiation for all patients regardless of their immunological status. Although these findings are not novel and have been observed in other PWH in France^27^ and elsewhere, our study documents that these guidelines were also readily implemented in TWH. However, the emergence of disparities based on geographic origin in the most recent period is of particular concern. In 2013–2023, TWH from Latin America and countries outside France were, respectively, 32% and 35% less likely to start ART early than those born in France, despite a healthcare system designed to provide universal access. These inequities likely reflect the time required to obtain proper housing, legal documents, and access to medical care for foreign-born TWH. A recent community-based study conducted in TWH in France (2020–2022) found that 33% struggled with unstable housing, 30% were undocumented migrants, and 24% were covered by the State Medical Aid (AME) while 5% had no health insurance, underscoring the structural barriers faced by this population to access HIV care.^20^ Although French policy has theoretically guaranteed access to HIV care for all since 1996, including undocumented migrants, and was further supported by the AME in 2000,^29^ the introduction of a three-month residency requirement in 2003 may have limited timely access to care for recently arrived migrants. However, our results showed that disparities by geographic origin only emerged after 2012, concurrently with implementation of the ‘treat all’ guidelines, suggesting that prior to this period, inequities in access to care were likely masked by immunological criteria that delayed ART initiation for all patients. Given that early ART initiation is paramount for HIV transmission control, clinical guidelines alone are insufficient. Additional efforts targeting the social determinants of health are needed to ensure equitable and timely access to HIV care for foreign-born TWH.

Over the 27-year study period, approximately 25% of TWH were lost to follow-up (LTFU), with a downward trend in recent years. This significant proportion does not necessarily indicate disengagement from care, as some TWH may have been receiving care in centers not included in the FHDH cohort, or in other countries. TWH in France represent a socially vulnerable population, with 77% foreign-born in our study, and a substantial proportion of TWH reporting transactional sex and unstable housing.^20^ Frequent mobility within European countries and between Europe and countries of origin is likely and may contribute to fragmented care pathways and interrupted follow-up. Structural and administrative barriers may further disrupt continuity of care.

In our study, the proportion of TWH on ART with viral suppression improved over time, reaching the UNAIDS 95% target in 2013–2023 (Fig. 3), a higher rate than previously reported in TWH in other geographical settings.^8^^,^^14^^,^^30^, ^31^, ^32^, ^33^, ^34^^,^^35^ Viral suppression at last follow-up remained slightly lower among TWH than among the overall population of PWH in the FHDH cohort (92% vs 97%, respectively).^36^ Comparisons across studies are limited by different viral suppression definitions, often based on a single VL < 200 or <50 copies/mL. Similar improvements were observed in North America, where viral suppression increased from 42% in 2007 to 73% in 2016 in New York City,^26^ and from 36% in 2001 to 80% in 2015 across the US and Canada,^8^ as well as in the Netherlands, where 87% of TWH achieved viral suppression in 2021.^14^ Compared with studies conducted among TWH in Latin America, HIV care outcomes in Latin American TWH in France are more favorable, likely reflecting a higher income context with more optimal access to HIV care and lower structural barriers.^30^^,^^31^^,^^35^ Suppression rates in TWH in France tend to be higher, as also reported in a community-based study where 94% of TWH had a VL < 200 copies/mL,^20^ possibly reflecting differences in healthcare systems and/or sampling methods. Across studies, the gap between TWH and other PWH appears to be narrowing over time, although TWH sometimes remain less likely to achieve virologic success.^9^^,^^12^^,^^13^ Further studies are needed to assess whether these patterns are observed in Europe.

This study is the largest and longest longitudinal analysis of TWH in care in Europe to date and, to our knowledge, the first to comprehensively examine delays along the HIV care continuum and their determinants in this key population over almost 3 decades. The use of a large, nationally representative cohort spanning multiple treatment eras allows for robust assessment of long-term trends and policy impacts.

There are several limitations to this study. First, some participants may have been missed in the earlier periods due to potential misclassification, as transgender identity could not be reliably identified before 2013. Nevertheless, this is likely attenuated as clinicians more frequently documented gender identity disorders before 2013, suggesting this was a way for them to indicate the transgender identity in medical records (Supplementary Figure S1). Second, our study is limited to TWH in care in hospitals, and does not account for those followed outside hospitals or not engaged in care, limiting generalizability to all TWH. Lastly, socioeconomic data that could explain disparities between migrant and non-migrant TWH are not collected in FHDH and therefore could not be analyzed to provide a deeper exploration of the mechanisms underlying observed disparities. These factors require specific investigation, as shown in the TRANS&HIV study,^20^ where lack of a gender-concordant identity document or health insurance was associated with virologic failure, reflecting broader inequalities in HIV outcomes.

TWH represent a key population facing intersecting social and structural barriers, particularly among migrants, which hinder equitable HIV care. Our findings and recent community-based evidence in France support the need for structural, healthcare, and community-based interventions to address barriers faced by TWH. These may include immediate access to HIV care and ART for migrants, better HIV care coordination across European countries, and improved access to legal residency and gender-concordant identity documents. Integrating transgender-inclusive care into HIV healthcare training and strengthening community-based support may further improve engagement and retention in care.

Moreover, in the coming years, the introduction of long-acting injectable ART and PrEP may represent a further shift in the organization of HIV care, moving beyond universal treatment initiation toward treatment continuity. This holds great promise particularly for key populations such as TWH, but will require careful attention to the risks of loss to follow-up and interruptions in care.

### Conclusion

In this nationwide study, HIV care and outcomes among TWH improved markedly over nearly three decades, with rapid ART initiation and viral suppression reaching the UNAIDS 95% targets in recent years. While the “treat-all” recommendations have contributed to faster ART initiation, emerging disparities based on geographic origin highlight the persistence of structural barriers and the need to implement targeted strategies to ensure equitable and rapid access to HIV care for all TWH.

## Contributors

PT, PdT and SG initiated the project. JH, EM and SG designed the study. JH analyzed the data. JH and SG had full access to the data, verified the data, and had final responsibility for the decision to submit the study for publication. JH and SG drafted the manuscript. All authors were involved in the interpretation of the data and critical revision of the manuscript and approved the final version.

## Data sharing statement

The data that support the findings of this study are available from the corresponding author upon reasonable request and access to the data on a secure platform can be given after approval from the French data protection agency (CNIL), and the ANRS CO4 FHDH scientific committee.

## Declaration of interests

PT reports participation in an advisory board at Gilead Sciences. PdT and JG reports grants for workshops, conferences and travel from Gilead Sciences and MSD. TC reports consulting fees from Gilead Sciences, ViiV Healthcare, MSD and GSK. SG reports participation on Data Safety Monitoring Boards for ANRS and Institut Pasteur clinical trials. All other authors declare no competing interests.

## Appendix A Supplementary data

Supplementary Tables and Figures

Translated Abstract
