# Supplementary material for: The HIV care continuum and determinants of antiretroviral therapy initiation among transgender women in France (1997–2023): a longitudinal cohort study
Source: Lancet Reg Health Eur. 2026 Aug 20;69:101820. doi: 10.1016/j.lanepe.2026.101820 (PMC13524482; doi:10.1016/j.lanepe.2026.101820)
Supplement: Supplementary file 1 [file mmc1.docx]

**SUPPLEMENTARY MATERIAL**

**From article:**

**The HIV care continuum and determinants of antiretroviral therapy initiation among transgender women in France, 1997–2023: a longitudinal cohort study.**

Juliette Hemery, Pierre Tattevin, Esaïe Marshall, Margot Annequin, Thibault Chiarabini, Nathalie Dournon, Anaenza Freire Maresca, Jade Ghosn, Valentina Isernia, Florence Michard, Segolene Perrineau^6^, Giovanna Rincon, Elisabeth Rouveix, Bruno Spire, Pierre de Truchis, Sophie Grabar

Contents

[Supplementary Table 1. Country of birth of TWH from Latin America included between 1997 and 2023 in the FHDH cohort (N= 522).	2](#_Toc234165424)

[Supplementary Table 2. Characteristics of TWH retained in care and those LTFU.	3](#_Toc234165425)

[Supplementary Table 3. Sensitivity analyses of ART uptake and viral suppression among TWH	5](#_Toc234165426)

[Supplementary Figure 1. Identification of transgender participants in the ANRS CO4 FHDH cohort.	6](#_Toc234165427)

[Supplementary Figure 2. Identification of the gender identity of transgender participants (TW/TM)^1^ in the ANRS CO4 FHDH cohort.	7](#_Toc234165428)

# Supplementary Table 1. Country of birth of TWH from Latin America included between 1997 and 2023 in the FHDH cohort (N= 522).

| **Country of birth** | **N (%)** |
| --- | --- |
| Unknown | 44 (8.3) |
| Peru | 223 (42.7) |
| Brazil | 109 (20.8) |
| Ecuador | 80 (15.3) |
| Colombia | 25 (4.8) |
| Argentina | 18 (3.4) |
| Venezuela | 12 (2.3) |
| Bolivia | 2 (0.4) |
| Guyana | 2 (0.4) |
| Panama | 2 (0.4) |
| Chile | 1 (0.2) |
| Costa Rica | 1 (0.2) |
| Paraguay | 1 (0.2) |
| Suriname | 1 (0.2) |
| Uruguay | 1 (0.2) |

Abbreviations: TWH, transgender women living with HIV; FHDH, French hospital database on HIV.

# Supplementary Table 2. Characteristics of TWH retained in care and those LTFU.

|  | **Not LTFU**  **(n=604, 75.4%)** | **LTFU**  **(n=197, 24.6%)** | **P value** |
| --- | --- | --- | --- |
| **Period of inclusion^1^** |  |  | **<0.0001** |
| 1997–2004 | 136 (22.5%) | 68 (34.5%) |  |
| 2005–2012 | 172 (28.5%) | 73 (37.1%) |  |
| 2013–2023 | 296 (49.0%) | 56 (28.4%) |  |
| **Age at inclusion** |  |  | **0.0027** |
| 18–29 | 232 (38.4%) | 99 (50.3%) |  |
| 30–39 | 264 (43.7%) | 79 (40.1%) |  |
| >40 | 108 (17.9%) | 19 (9.6%) |  |
| **Geographical origin** |  |  | 0.4416 |
| Africa | 33 (5.5%) | 11 (5.6%) |  |
| France | 143 (23.7%) | 39 (19.8%) |  |
| Latin America | 385 (63.7%) | 137 (69.5%) |  |
| Other countries | 43 (7.1%) | 10 (5.1%) |  |
| **Region of inclusion** |  |  | 0.1792 |
| Paris region | 414 (68.5%) | 145 (73.6%) |  |
| Other regions | 190 (31.5%) | 52 (26.4%) |  |
| **Immunological status at enrolment** |  |  | 0.4764 |
| <200 CD4 / AIDS | 132 (21.9%) | 51 (25.9%) |  |
| 200–350 CD4 | 123 (20.4%) | 36 (18.3%) |  |
| >350 CD4 / primary infection | 349 (57.8%) | 110 (55.8%) |  |
| **First cART regimen** |  |  | 0.7035 |
| Dual | 19 (3.2%) | 7 (3.7%) |  |
| Triple therapy | 583 (96.8%) | 181 (96.3%) |  |
| **HIV RNA (cp/mL)** |  |  | 0.1295 |
| <5000 | 76 (12.6%) | 30 (15.2%) |  |
| 5 000–100 000 | 287 (47.5%) | 104 (52.8%) |  |
| ≥100 000 | 241 (39.9%) | 63 (32.0%) |  |
| **Hep B** | 21 (3.5%) | 9 (4.6%) | 0.4834 |
| **Hep C** | 17 (2.8%) | 3 (1.5%) | 0.3130 |
| **Duration of fwup (years)** | 10.6 (5.1 – 17.3) | 8.6 (3.9 – 13) | **0.0001** |
| **ART initiation during follow-up** | 602 (99.7%) | 188 (95.4%) | **<.0001** |
| **Viral suppression^2^ during follow-up** | 554 (94.1%) | 160 (81.6%) | **<.0001** |

P-value from chi-square or Kruskal-Wallis tests comparing TWH retained in care and those LTFU.

^1^N (%) or median (IQR).

^2^Viral suppression was defined as two consecutive viral load measurements <200 copies/mL.

Abbreviations: TWH, transgender women living with HIV; ART, antiretroviral therapy.

# Supplementary Table 3. Sensitivity analyses of ART uptake and viral suppression among TWH.

| **Periods** | **1997–2004** | | **2005–2012** | | **2013–2023** | |
| --- | --- | --- | --- | --- | --- | --- |
|  | **ART** | **VS^1^** | **ART** | **VS^1^** | **ART** | **VS^1^** |
| **Main analysis N=801 (fig 3)** | 81.1% | 74.7% | 93.5% | 93.6% | 99.4% | 95.5% |
| **Sensitivity analysis 1^*^**  **N=910** | 80.5% | 75.2% | 92.9% | 91.9% | 99.2% | 95.0% |
| **Sensitivity analysis 2****  **N= 966** | 79.7% | 82.3% | 91.7% | 93.6% | 96.2% | 94.8% |

^1^VS: Viral suppression achievement

^*^Sensitivity analysis 1: we included TWH with missing baseline CD4/VL data (n=81+16), UDI (n=10), and atypical ART regimens (n=2), for a total sample of 910 TWH while keeping our original definition of viral suppression (two VL measurements <200 copies/mL).

^**^Sensitivity analysis 2: we included all previously excluded TWH (n=81+16+10+2), as well as those with only one VL after ART initiation (n=56) for a total sample of 966 TWH. Given that only one VL measurement was available for 56 TWH, we modified the definition for viral suppression: achievement of one VL<200 cp/mL instead of two consecutive VL<200.

Abbreviations: TWH, transgender women living with HIV; ART, antiretroviral therapy.

# Supplementary Figure 1. Identification of transgender participants in the ANRS CO4 FHDH cohort.

PWH adults enrolled in the ANRS CO4 FHDH cohort between 01/01/1997 and 12/31/2023

**N= 160,927**

**Gender based on the variable collected in the FHDH cohort since 2013**

N= 1686 transgender

N= 57,173 female

N= 102,068 male

N=239 participants with ICD-10 diagnosis code ‘F64’ for "*gender identity disorder*"^1^
(N= 220 male and N= 19 female)

**Recoded gender used for the analyses**

N= 57,154 female

N= 1925 **transgender**:

N= 220 TW

N= 19 TM

N= 1686 unknown

N= 101,848 male

Note: TW: transgender women, TM: transgender men.

^1^Among the 239 participants diagnosed with ICD-10, 183 were diagnosed before 2013, and 56 after 2013.

# Supplementary Figure 2. Identification of the gender identity of transgender participants (TW/TM)^1^ in the ANRS CO4 FHDH cohort.

**Transgender adults** enrolled in the ANRS CO4 FHDH cohort
between 01/01/1997 and 12/31/2023

**N= 1925**

N= 220 TW

N= 19 TM

N= 1686 unknown

“Men who have sex with men” (MSM) as mode of HIV acquisition (n= 1535)

N= 1755 TW

N= 19 TM

N= 151 unknown

Sex-specific malignant neoplasms of the male genital organs (n=6) or female organs (n=3) using ICD-10 code 'C60' to 'C63' and 'C51' to 'C58' respectively.

N= 1761 TW

N= 22 TM

N= 142 unknown

Receiving feminizing hormone therapy (n=20) or masculinizing hormone therapy using ATC code^2^ (n=0).

N= 1781 (92.5%) Transgender women

N= 22 (1.1%) Transgender men

N= 122 (6.3%) unknown

**Recoded gender used for the analyses**

^1^TW: Transgender women (Male to female) / TM: Transgender men (Female to Male)

^2^ATC codes: 'G03CA03' (estradiol), 'G03HB01'/'G03HA01' (cyproterone) for feminizing hormone therapy. And 'G03BA03' (testosterone) for masculinizing hormone therapy.
