# Supplementary material for: The HIV care continuum and determinants of antiretroviral therapy initiation among transgender women in France (1997–2023): a longitudinal cohort study
Source: Lancet Reg Health Eur. 2026 Aug 20;69:101820. doi: 10.1016/j.lanepe.2026.101820 (PMC13524482; doi:10.1016/j.lanepe.2026.101820)
Supplement: Supplementary file 2 [file mmc2.docx]

**Abstract in FRENCH :**

***Disclaimer:*** *This translation in French was submitted by the authors and we reproduce it as supplied. It has not been peer reviewed. Our editorial processes have only been applied to the original abstract in English, which should serve as reference for this manuscript.*

**Le continuum de soins et les déterminants de l’initiation des traitements antirétroviraux chez les femmes transgenres vivant avec le VIH en France, 1997–2023 : une étude longitudinale de la cohorte ANRS CO4 FHDH.**

**Juliette Hemery^1^**, Pierre Tattevin^2^, Esaïe Marshall^1^, Margot Annequin^3^, Thibault Chiarabini^4^, Nathalie Dournon^5,6,7^, Anaenza Freire Maresca^8,9^, Jade Ghosn^10,11^, Valentina Isernia^10^, Florence Michard^10^, Segolene Perrineau^6^, Giovanna Rincon^12^, Elisabeth Rouveix^6,13^, Bruno Spire^3^, Pierre de Truchis^5,7,13^, Sophie Grabar^1,4^.

**Contexte :** Les données concernant les femmes transgenres vivant avec le VIH (FTVIH) en Europe sont limitées. Cette étude avait pour objectif de décrire leur continuum de soins en France sur une période de plus de 25 ans (1997 – 2023), en évaluant les déterminants associés au délai d’initiation des traitements antirétroviraux (ARV).

**Méthodes :** A partir de la cohorte ANRS CO4-FHDH (base de données hospitalière française sur le VIH), qui couvre près de 65% de personnes vivant avec le VIH prises en charge en France, nous avons sélectionné les FTVIH naïves de traitement ARV incluses entre le 1^er^ janvier 1997 et le 31 décembre 2023. Les indicateurs du continuum de soins du VIH, tels que la proportion sous ARV et ayant atteint une suppression virale, ainsi que les délais entre les étapes clés du continuum (diagnostic du VIH – prise en charge – initiation des ARV – suppression virale) ont été évalués sur trois périodes calendaires : 1997–2004, 2005–2012 et 2013–2023. Les facteurs associés au délai d'initiation des ARV ont été analysés pour chaque période à l'aide de modèles de Cox ajustés sur l'âge, l'origine géographique, l'année et la région d'inclusion, le délai entre le diagnostic et l'inclusion, ainsi que le statut immuno-virologique initial.

**Résultats :** Un total de 801 FTVIH ont été incluses entre 1997 et 2023. La majorité était originaire d’Amérique latine (65 %), et l’âge médian était de 31 ans [IQR 27–36]. Entre 1997–2004 et 2013–2023, le délai d’initiation des ARV après la prise en charge a diminué de 3,9 [0–31] à 0,5 [0,2–1,2] mois. Le statut immunologique était fortement associé au délai d’initiation des ARV jusqu’en 2012. En 2013–2023, une charge virale initiale ≥100 000 copies/mL était associée à une initiation plus rapide des ARV (HR: 1,45 ; IC95 %: 1,14–1,86 ; p=0,003), tandis qu’être originaire d’Amérique latine (HR: 0,68 ; IC95 %: 0,50–0,91) ou d’autres pays en dehors de la France (HR: 0,65 ; IC95 %: 0,45–0,95) était associé à un retard d’initiation des ARV (p global = 0,022). Entre 1997–2004 et 2013–2023, le délai entre l’initiation des ARV et la suppression virale a diminué de 6,2 [3,0–27,9] à 2,3 [1,1–5,7]. Sur les mêmes périodes, la proportion de FTVIH sous ARV et avec une suppression virale est passée de 74,7 % à 95,5 %.

**Interprétation :** Malgré des améliorations majeures du continuum de soins chez les FTVIH en France, avec une initiation plus rapide des ARV et un taux de suppression virale atteignant les objectifs de l’ONUSIDA de 95 % en 2013–2023, des inégalités selon l’origine géographique ont émergé, avec un retard d’initiation des ARV chez les FTVIH nées à l’étranger. Etant donné que la majorité de cette population clé est née à l’étranger, des stratégies et politiques ciblées sont nécessaires pour garantir un accès équitable et rapide aux soins liés au VIH.

**Mots-clés :** France, VIH, continuum de soins VIH, initiation des ARV, femmes transgenres, suppression virale.

**Financement** : Cette recherche a été financée par l’ANRS-MIE and INSERM.
